# Daptomycin-Induced Severe Hyperkalemia With Normal Creatine Kinase in a Patient With Methicillin-Resistant Staphylococcus aureus Osteomyelitis

**DOI:** 10.7759/cureus.44674

**Published:** 2023-09-04

**Authors:** Kiranpreet K Sawhney, Fatai Oluyadi

**Affiliations:** 1 Internal Medicine, Edward Via College of Osteopathic Medicine - Carolinas Campus, Spartanburg, USA; 2 Internal Medicine, Medical University of South Carolina - Lancaster, Lancaster, USA

**Keywords:** methicillin-resistant staphylococcus aureus ( mrsa), osteomyelitis, creatine kinase (ck), hyperkalemia, daptomycin

## Abstract

We report a case of an asymptomatic 60-year-old female who presented to the emergency department due to a home health measured serum potassium of 7.7 mmol/L (normal range: 3.6-5.0 mmol/L) and was admitted for severe hyperkalemia. She was recently started on a low dose of daily intravenous daptomycin to treat methicillin-resistant *Staphylococcus aureus* (MRSA) osteomyelitis of her sacral decubitus ulcers. Laboratory results showed normal creatine kinase (CK). Her elevated serum potassium levels reversed throughout her hospital stay and remained within normal range after daptomycin discontinuation, establishing a temporal relationship between daptomycin and hyperkalemia. To our knowledge, no other cases report daptomycin-induced severe hyperkalemia in the absence of rhabdomyolysis. Our case emphasizes the importance of considering hyperkalemia as an adverse effect of daptomycin, especially in elderly hospitalized patients.

## Introduction

Hyperkalemia is a common and clinically important electrolyte abnormality. It is classified as mild (5.5 to 6.5 mmol/L), moderate (6.5-7.5 mmol/L) and severe (> 7.5 mmol/L) hyperkalemia based on the serum potassium levels [1]. Although mild hyperkalemia is asymptomatic, symptoms begin to develop when serum potassium is > 6.5 mmol/L. More commonly, it manifests as muscle weakness, paralysis, and relatively benign changes on electrocardiogram, i.e., peaked T waves, prolonged PR-intervals, and widened QRS complexes. Moderate to severe hyperkalemia, however, is a medical emergency primarily due to life-threatening cardiac abnormalities like bradyarrhythmia, heart blocks, ventricular fibrillation, or asystole [2,3]. Increased risk of mortality related to hyperkalemia has been well established not only in patients with chronic kidney disease (CKD) [4], but also in patients with normal kidney function [3].

Causes of true hyperkalemia are diverse; however, they essentially originate from either increased potassium intake, impaired potassium excretion, or a cellular-level disturbance in potassium homeostasis [5]. While renal insufficiency (i.e., acute kidney injury (AKI), CKD) is a well-known risk factor, diabetes mellites (DM), hypoaldosteronism, congestive heart failure (CHF) and massive tissue breakdown (i.e., rhabdomyolysis, hemolysis) are also notable conditions that result in hyperkalemia [1,6].

Medications are a frequent cause of hyperkalemia, especially for the elderly in hospital settings [6]. The most common drugs associated with an adverse drug reaction (ADR) of hyperkalemia are potassium-sparing diuretics (i.e., spironolactone), angiotensin-converting enzyme inhibitors (ACEI), angiotensin II receptor blockers (ARBs), non-steroidal anti-inflammatory drugs (NSAIDs), beta-blockers (BB) and potassium-chloride supplement [1,5,6]. Although antibiotic-induced hyperkalemia is unusual, it has most commonly been reported as an ADR of trimethoprim/sulfamethoxazole [6].

Daptomycin is an uncommon, but another possible antibiotic to induce hyperkalemia. Typically, hyperkalemia has been noted with rhabdomyolysis, which is the more commonly associated adverse effect of daptomycin [7,8]. It is thought that muscle cell necrosis (that causes rhabdomyolysis) initiates the release of intracellular ions such as potassium, leading to correlated hyperkalemia. There have been three case reports highlighting daptomycin-induced hyperkalemia [7-9]. While two of the case reports explicitly associate hyperkalemia with daptomycin-induced rhabdomyolysis [7,8], one attempts to dissuade such an association [9]. Here, we present a case of severe hyperkalemia with normal levels of creatine kinase (CK) that occurred 11 days after the initiation of daily IV daptomycin 6 mg/kg (400 mg).

## Case presentation

A 60-year-old asymptomatic African American female presented to the emergency department (ED) due to elevated serum potassium of 7.7 mmol/L as noted by the home health nurse and was admitted for severe hyperkalemia. She has a past medical history of stroke with severe left-sided weakness, coronary artery disease (CAD) with a stent, CHF, and peripheral vascular disease (PVD) status post right above-knee amputation (AKA). A month ago, she was hospitalized for sepsis due to stage IV sacral decubitus ulcers that were complicated by methicillin-resistant *Staphylococcus aureus* (MRSA) osteomyelitis and a urinary tract infection with multi-drug resistant Escherichia coli. During her hospital stay, she was found to have a sacral abscess, which was surgically drained. She was initially treated with 1 g of IV vancomycin every 12 hours and 1 g of meropenem every eight hours. Vancomycin was discontinued 10 days into treatment due to AKI with a serum creatinine elevated at 1.5 mg/dL and consequent hyperkalemia. Notably, her random vancomycin trough levels peaked at 15.4 mg/L (reference range: 10-20 mg/L). She was discharged, 11 days prior to the current ED visit, with wound care and home infusion of a daily low dose of IV daptomycin 6 mg/kg (400 mg) and 1 g of meropenem every eight hours. She was on atorvastatin 80 mg daily; however, that medication was discontinued before daptomycin initiation. Her home medication list is provided in Table 1.

**Table 1:** The patient’s home medication list at the time of admission. * Patient taking a different dose than prescribed (ASA 325 mg)

Medication or Supplement	Dosage	Frequency and Route of Administration
Acetaminophen	325 mg	2 tablets PO every 4 hours PRN
Albuterol	90 mcg	1-2 puffs every 4 hours PRN
Amlodipine	5 mg	Once daily PO
Aspirin	81mg*	Once daily PO
Chlorhexidine gluconate	15 mL	Take orally once at bedtime
Collagenase	250 units/gram	Apply topically daily
Daptomycin	400 mg	Intravenous administration once daily for 15 days
Ferrous sulfate	325 mg	Once daily PO
Insulin detemir	100 unit/mL	Inject 10 units subcutaneously at bedtime
Meropenem	1 g	Intravenous administration every 8 hours for 14 days
Ondansetron	4 mg	Sublingual tablet every 8 hours PRN
Oxycodone	30 mg	Once daily PO
Oxycodone IR	15 mg	1 tablet PO every 4 hours PRN

Upon presentation, she denied any symptoms of chest pain, palpitations, shortness of breath, dizziness, constipation, diarrhea, and abdominal pain. Her vitals, however, were significant for elevated blood pressure at 157/74 and tachycardia with a pulse of 111 beats per minute. Overall, she was alert and oriented. On physical exam, she had multiple dressed sacral and gluteal wounds, as well as a dressed left ankle wound. Her neurological exam demonstrated residual left lower extremity weakness from her previous stroke. The results of the complete metabolic panel (CMP) and CK laboratory tests ordered throughout the hospital stay are summarized in Table 2.

**Table 2:** The complete metabolic panel (CMP) and creatine kinase (CK) laboratory results throughout the three-day hospital stay. Blood urea nitrogen (BUN), estimated glomerular filtration rate (eGFR) * Normal range of creatine kinase (CK) for females † Discontinuation of daily IV daptomycin 6 mg/kg

	Day 1 (16:00)	Day 1 (23:25)	Day 2 (1:02)	Day 2 (11:47)	Day 3 (00:47)	Day 3 (15:25)	Reference range/units
Sodium	134	136	136	137	137		135 – 145 mmol/L
Potassium, serum	7.70	7.30	7.00	6.00	5.20†	4.70	3.60 – 5.00 mmol/L
Chloride	102	105	105	102	105		101 – 111 mmol/L
CO2	25	23	24	23	25		21 – 31 mmol/L
Anion Gap	14.7	15.3	14	18.0	12.2		8.0 – 16.0 mmol/L
Glucose	136	114	108	93	95		70.0 – 100.0 mg/dL
BUN	33	34	34	30	26		7 – 18 mg/dL
Creatinine	0.80	0.80	1.10	0.78	0.80		0.50 – 1.20 mg/dL
eGFR, African American	89	89	61	91	89		> 89 mL/min
Calcium	9.0	9.1	9.0	9.0	8.6		8.4 – 10.2 mg/dL
Creatine Kinase	53			46			10 – 70 U/L*

Serum potassium was noted to be elevated at 7.7 mmol/L on presentation, and 7.3 mmol/L on admission to the ED. Of note, serum potassium was 4.0 mmol/L when IV daptomycin and meropenem were initiated. Additionally, upon presentation, creatinine was 0.8 mg/dL, BUN was elevated at 33 mg/dL, eGFR was stable at 89 and the anion gap was within the normal range of 12-16. After admission, creatinine rose to 1.1 mg/dL, BUN remained elevated at 34 mg/dL, eGFR dropped to 61 and the anion gap rose to 18, indicating a metabolic acidosis. The elevated BUN, and the 0.3 mg/dL rise in creatinine within a 24-hour time frame supported a diagnosis of mild AKI. Given the recent history of daptomycin initiation and the above lab abnormalities, there was a significant concern for rhabdomyolysis. The CK was noted to be 53 U/L (reference range: 10-70 U/L) upon presentation and 46 U/L the following day, which ruled out the possibility of rhabdomyolysis. An electrocardiogram showed sinus tachycardia and probable left atrial enlargement; however, there were no peaked T-waves or widened QRS complexes noted.

Of note, the patient’s baseline potassium a few days before the ED visit was 5.1 mmol/L, which is borderline for elevated serum potassium. However, her rise in serum potassium was pronounced, peaking at 7.7 mmol/L, hence classifying this event as severe hyperkalemia. Given her severely elevated serum potassium on presentation, the patient was treated with the hyperkalemia management protocol including intravenous insulin with dextrose 50% solution and calcium gluconate. After treatment, serum potassium was measured to be 7.0 mmol/L. Since the potassium levels were still elevated, the hyperkalemia management therapies were repeated with the addition of oral Kayexalate and intravenous furosemide. She was also started on sodium bicarbonate to correct the metabolic acidosis and intravenous fluids (IVF) to resolve the mild AKI. A foley catheter was inserted to strictly monitor the inputs and outputs. On day 3, total output was noted to be 2,850 mL over a 24-hour period, which is about 1.45 mL/kg/hour. Daptomycin was discontinued early on hospital day 3, and the patient was discharged with IV vancomycin for 10 days to complete the full course of six-week antibiotic treatment of MRSA osteomyelitis. No new episodes of hyperkalemia were noted after daptomycin was discontinued.

## Discussion

Daptomycin is a cyclic lipopeptide antibiotic used primarily in the treatment of resistant gram-positive microorganisms such as MRSA and vancomycin-resistant enterococci (VRE) among others [10]. Commonly, it treats complicated skin and soft tissue infections (cSSTIs), right-sided endocarditis, and bacteremia [11]. It is also an effective and safe alternative in treating refractory MRSA infections of the bone, joints, and prosthetic devices [12]. Since daptomycin has a lower and less severe risk of AKI than vancomycin, it is a good alternative option for patients with reduced kidney function [13].

Daptomycin is a bactericidal antibiotic. It works by disrupting the bacterial cell membrane structure with its lipid tail, which forms holes that lead to the loss of ions, and thus, loss of membrane potential and subsequently, cell death [11]. While daptomycin is generally well tolerated, it can precipitate rhabdomyolysis, which is the breakdown of muscle tissue, in a dose-dependent manner [10]. At higher doses of daptomycin (8 mg/kg/day), the disruption of skeletal muscle integrity causes an efflux of intracellular components and ions (i.e., potassium) to spill into the extracellular compartment, causing hyperkalemia [14].

Hyperkalemia is a less established adverse effect of daptomycin. Although there are three previously reported cases of daptomycin-induced hyperkalemia, each case occurs in the presence of rhabdomyolysis [7-9]. The most recent case highlights how the concurrent use of daptomycin and atorvastatin can induce rhabdomyolysis, which is mediated by severe hyperkalemia and AKI [8]. Another relatively recent case introduces hyperkalemia as an early indicator of daptomycin-induced rhabdomyolysis [7]. Both cases recognize rhabdomyolysis as the primary ADR to daptomycin and establish an association between hyperkalemia and rhabdomyolysis [7,8]. The earliest known case of daptomycin-induced hyperkalemia, however, claims hyperkalemia and rhabdomyolysis to be independent adverse reactions of daptomycin. In this case, rhabdomyolysis was presented earlier (on day 10), while the first elevation in serum potassium was noted later (on day 14) [9]. This is an unusual temporal relationship between hyperkalemia and rhabdomyolysis and thus is argued to indicate a lack of association between the two outcomes. However, since hyperkalemia has been described as an early presentation and an early complication of rhabdomyolysis, it is difficult to completely ignore the potential contribution of rhabdomyolysis to the rise in serum potassium [7,8,15]. 

The current case describes a striking incidence of severe hyperkalemia in the absence of rhabdomyolysis induced 11 days post-daptomycin treatment initiation. The patient notably did not have a history of kidney disease, which can cause higher levels of potassium to accumulate in the blood. She also had relatively stable kidney function throughout the hospital stay, except for stage one AKI, which may have contributed to hyperkalemia. However, that does not explain the severity of the hyperkalemia as patients with AKI usually experience modest rises in their serum potassium [16]. Additionally, hyperkalemia is known to be a common complication, particularly, in oliguric AKI due to the reduced ability to excrete the excess potassium. In this case, however, the patient was able to produce urine at levels greater than 0.5 mL/kg/hour.

The patient’s home medications provided in Table 1 show that she was not on medications known to precipitate hyperkalemia, i.e., potassium-sparing diuretics, ACEI, ARBs, BB, or potassium chloride supplements [5,6]. She was on amlodipine, which is a calcium channel blocker (CCB), for the treatment of hypertension. Although CCBs have some association with hyperkalemia, it is more commonly noted with Nifedipine [5]. Also, after the patient was admitted to the hospital, she was started on heparin for deep vein thrombosis prophylaxis. Heparin is also associated with hyperkalemia, however, given that heparin was initiated after the peak hyperkalemic event, it is unlikely to be the cause. This process of elimination in conjunction with the absence of hyperkalemia events post-daptomycin discontinuation supports daptomycin as the most likely originating source of severe hyperkalemia. Also of note, the time from daptomycin administration to the first event of hyperkalemia, in this case, was 11 days, which is similar (ranging from 10 to 14 days) across most reported cases [7,8]. The ADR probability scale established by Naranjo et al. yielded a score of 6, indicating that daptomycin was a probable cause of the severe hyperkalemia noted in this case [17].

It is important to recognize hyperkalemia as an ADR of daptomycin. Although it is not a common ADR, it should be given with caution in patients at a greater risk for drug-induced hyperkalemia. Significant risk factors associated with medication-related hyperkalemia include polypharmacy (>5 drugs), age (> 60), renal insufficiency (glomerular filtration rate <60 mL/min), and the presence of ≥ 4 comorbid conditions (i.e., DM, hypertension, kidney disease, hypoaldosteronism) [5,6]. Surveilling for the above risk factors can help reduce the occurrence and severity of hyperkalemic events in the hospital setting. According to a prospective study, roughly a third of severe hyperkalemia events recorded in a hospital setting were attributed to medications [6]. Surprisingly, of all the drug-induced hyperkalemic episodes in older hospitalized patients, 79.9% of them were avoidable [18]. While it is an alarming statistic, it is also a hopeful finding, suggesting that there is potential to reduce drug-induced hyperkalemia and hyperkalemia-related complications by being aware of and incorporating the known risk factors into our management of hyperkalemia.

There are some notable limitations in this case. Regarding labs, while the complete blood count, CMP, CK, and strict inputs and outputs were followed, liver enzymes, aldosterone levels, renin levels, serum lactate, and serum uric acid among other labs were not measured. Also, pseudo-hyperkalemia, which is hyperkalemia that results from the leakage of potassium during or after blood sampling, was not accounted for [1]. Given that potassium was rechecked multiple times throughout the hospital stay, this would not be reason enough to ignore the hyperkalemic event. It is also important to note that although the patient has a higher default potassium, her rise in serum potassium was pronounced. There was an increase of 2.6 mmol/L over her baseline, leading to a severe hyperkalemic event measured at 7.7 mmol/L.

A severe hyperkalemic event with normal creatine kinase was noted 11 days after initiation of daily IV daptomycin 6 mg/kg (400 mg). Although stage one AKI and metabolic acidosis were noted, they do not explain the severity of hyperkalemia in a non-oliguric patient with an estimated glomerular filtration rate > 60. Similarly, the home medications were not significant for having hyperkalemia as an ADR. Post discontinuation of daptomycin, there were no elevations of serum potassium noted. Since there is a temporal relationship between daptomycin and hyperkalemia in the absence of another plausible explanation, daptomycin is the most probable factor for the isolated severe hyperkalemic episode in our patient.

## Conclusions

Although Daptomycin is considered a safer option than Vancomycin for MRSA infections in patients with renal insufficiency, it also has its own risks. This unique case highlights a severe probable daptomycin-induced hyperkalemic event in a patient without rhabdomyolysis or significant renal impairment. It encourages us to consider the possibility of hyperkalemia as an independent ADR of daptomycin, especially in elderly hospitalized patients with an increased risk of drug-induced hyperkalemia. It is worth considering further investigations to improve surveillance protocols and monitoring in hopes of reducing avoidable complications of medication-induced hyperkalemia.
